# A pilot study evaluating changes to haematological and biochemical tests after Flexible Ureterorenoscopy for the treatment of kidney stones

**DOI:** 10.1371/journal.pone.0179599

**Published:** 2017-07-06

**Authors:** Alyson Jayne Moyes, Rebecca May Lamb, Peter Ella-Tongwiis, Anish Pushkaran, Issam Ahmed, Iqbal Shergill, Stephen Fôn Hughes

**Affiliations:** 1 Department of Biological Sciences, University of Chester, Chester, United Kingdom; 2 North Wales & North West Urological Research Centre (NW2URC), Betsi Cadwaladr University Health Board (BCUHB) Wrexham Maelor Hospital, Wrexham, Wales, United Kingdom; 3 Department of Urology, BCUHB Wrexham Maelor Hospital, Wrexham, Wales, United Kingdom; 4 Department of Medical Sciences, Bangor University, Bangor, Wales, United Kingdom; Northwestern University, UNITED STATES

## Abstract

**Background:**

Currently there is limited research documenting the changes in blood parameters, following Flexible Ureterorenoscopy. This study aims to determine whether there are any changes in haematology and biochemistry parameters, following Flexible Ureterorenoscopy for the treatment of kidney stones.

**Methods:**

40 consecutive patients aged between 27–87 years (median 49 years) undergoing Flexible Ureterorenoscopy for the treatment of kidney stones were recruited (26 male, 14 female). Blood samples were collected from each patient at four time points: baseline (pre-operatively) followed by 30 minutes, 120 minutes and 240 minutes post-operatively. On these samples, routine haematological and biochemistry tests were carried out. In addition to the assessment of clinical parameters prospectively from the medical notes.

**Results:**

There was a significant decrease observed following Flexible Ureterorenoscopy in the following parameters: lymphocytes (p = 0.007), eosinophils (p = 0.001), basophils (p = 0.001), haemoglobin (p = 0.002), red blood cells (p = 0.001), platelet count (p = 0.001), fibrinogen concentration (p = 0.001), von Willebrand factor (p = 0.046), C reactive protein (p = 0.01), total protein (p = 0.001), albumin (p = 0.001), globulin (p = 0.001) and alkaline phosphatase (p = 0.001). In addition, there was a significant increase observed in the following parameters: white blood cells (p = 0.001), neutrophils (p = 0.001), activated partial thromboplastin time (p = 0.001), total bilirubin (p = 0.012), creatinine (p = 0.008), sodium (p = 0.002) and potassium (p = 0.001). Limiting factors for this study were the sample size, and restriction on the recruitment time points.

**Conclusions:**

Significant changes were noted to occur in haematology and biochemistry parameters following Flexible Ureterorenoscopy. Some of the data presented in this study may represent the ‘normal’ post-operative response following FURS, as no major complications occurred, in the majority of our patients. This data on the ‘normal response’ will need to be validated but may ultimately aid clinicians in distinguishing patients at risk of complications, if reproduced in larger multi-centre studies.

## Introduction

Kidney stones are increasing in prevalence and are recognised as a significant health issue [1], affecting 2–3% of the worldwide population [2]. In 2015, there were 49 million cases of kidney stones recorded, contributing to 15,000 deaths globally [3], [4]. Furthermore, it is predicted that 50% of patients who have had previous stones, will experience stone recurrence within 10 years [5]. Recent epidemiological analysis has highlighted a 63% increase in the number of hospital admissions in England, for kidney stones, during the past decade, with 86,742 cases reported in 2014–2015 alone [6]. Importantly, during this time there has been a 103% increase in the number of patients receiving ureteroscopic stone treatments since 2009/2010 [6]. This combination of escalating incident rates, high stone recurrence rates and increasing use of minimally invasive treatments, poses a considerable financial and economic burden for healthcare systems [7].

In the current European Association of Urology guidelines, Shock Wave Lithotripsy (SWL) and Flexible Ureterorenoscopy (FURS) are considered equally suitable treatments for stones between 11–20mm [8]. The Clinical Research Office of the Endourological Society (CROES) study found that treatment of single intrarenal stones <10 mm in diameter with FURS, resulted in a 90% successful stone free rate (SFR), and the SFR was 80% for stones as big as 15 mm [9]. As such, FURS has become the increasingly preferred treatment for kidney stones in most cases.

The overall complication rate after FURS is 9–25% [10], with the CROES URS global study identifying that bleeding, fever, Urinary Tract Infections (UTIs) and sepsis are common post-operative complications following FURS. With increasing incidence, high recurrence rates and annual increase in patients undergoing FURS, it is expected that the instances of such complications will increase [11].

The aim of this study was to evaluate haematological and biochemistry changes in patients undergoing FURS for the treatment of kidney stones. It was our expectation to enhance the current urological literature by adding evidence based insight on this topic, and to provide findings that may ultimately aid clinicians in distinguishing patients at risk of complications such as bleeding or infection following FURS.

## Materials and methods

### Subject volunteers and FURS

Ethical approval for this pilot study was received from the Welsh Research Ethics Service (REC) 4 committee (REC4: 12/WA/0117). Forty consecutive patients scheduled for elective FURS for the treatment of kidney stones were recruited at their pre-operative assessment after providing written informed consent (n = 40). The patients (26 male and 14 females) were aged between 27–87 years (median 49 years). Of the 40 patients, 27 had a single stone, 10 had two stones and 3 patients had 3 stones in the target kidney. The median stone size was 7.0 mm (range: 2.0 mm—25.0 mm). The median Hounsfield unit was 976 (range: 153–2224). The location of the stones was upper pole (n = 15), mid-pole (n = 8) and lower pole (n = 17). Treatment was given as per standardised protocol in our institution using the Olympus P5 Flexible Ureterorenoscope whilst under general anaesthesia using Propofol, with 75% (30/40) of the patients receiving laser lithotripsy during the procedure for a median duration of 14.58 minutes (range: 30 seconds– 54.02 minutes), at an average energy of 3232.7 J, and a mean pulse of 6272.76. The median operative time for the FURS procedure was 49 minutes (range: 22–104 minutes). Complete stone clearance was achieved in all patients (n = 40) with 25/40 of the patients requiring post-operative stent insertion. Stone samples for biochemical analysis were collected where possible, and the composition was analysed using infrared spectroscopy at the Leicester Royal Infirmary.

### Blood samples

On the day of their intervention, each patient had a venous blood sample collected from the arm, prior to the FURS procedure, which stood as a baseline (control) measurement. Following FURS, additional blood samples were collected at 30, 120 and 240 minutes post-operatively. Three vacutainers of blood were collected at each interval (di-potassium ethylene diamine tetra-aceticacid (EDTA), tri-sodium citrate and serum separator tube). All haematological and biochemical analysis was carried out on the day of the procedure on fresh whole blood/plasma, with exception of vWF analysis which was carried out in batches every 6 months on frozen patient plasma, stored at -80^°^C, in accordance to the manufacturers protocol. Subject plasma was obtained by centrifuging whole blood samples at 450g for 15 minutes.

### Measurement of full blood count and platelet concentration

Complete blood counts were performed using a Sysmex XE-5000 automated cell counter and platelets measured in units x10^9^/L.

### Measurement of activated partial thromboplastin time (aPTT), prothrombin time (PT), fibrinogen and von willebrand factor (vWF)

aPTT (seconds), PT (seconds) and Fibrinogen (g/L) concentrations were measured using citrated samples using the Sysmex CS2100 analyser. Plasma vWF concentrations (IU/dL) were measured in the frozen samples, using a two-step sandwich method, enzyme immunoassay (ELISA) using rabbit anti-human vWF and rabbit anti-human vWF peroxidase conjugate.

### Measurement of plasma viscosity (PV)

Plasma viscosity (mPa/second) was measured using a Benson BV200 viscometer.

### Measurement of biochemistry parameters

Biochemistry parameters were measured using the Beckman Coulter AU5800 and AU680 analyser.

### Statistical analysis

All results were presented as mean ± standard error (SE) or median ± min/max. Where data was normally distributed, repeated measures one-way analysis of variance (ANOVA) between samples test was employed, adopting a 5% level of significance. Post hoc testing was conducted using the Bonferroni test for pairwise comparisons between means. Data that did not comply with normality was analysed using the Friedman test. Where the Friedman test resulted in statistical significance, subsequent tests were performed using the Wilcoxon test. Statistical significance was accepted when p ≤ 0.05.

## Results

### Haematological blood results

**Fig 1:**
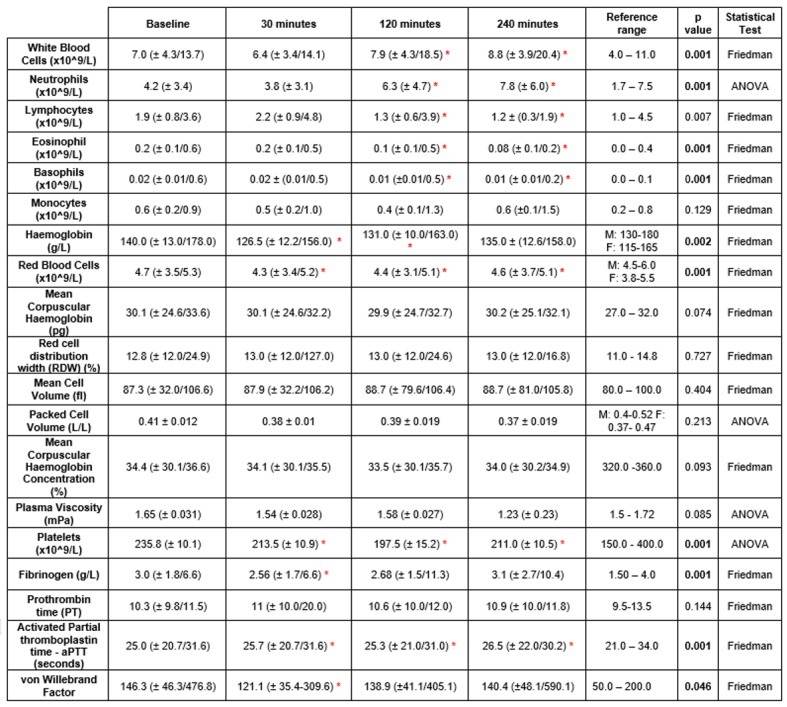
The effect of FURS, for the treatment of kidney stones on haematology parameters (n = 40). Data analysed via Friedman testing is presented as median ± minimum / maximum, data analysed via ANOVA is presented as means ± standard error. Statistical significance following post hoc analysis is represented when*p≤0.05. (M = male, F = female).

“Fig 1” demonstrates the changes in haematological parameters observed following FURS for the treatment of kidney stones (n = 40). Significant decreases were observed post FURS in the following parameters: lymphocytes (p = 0.007), eosinophils (p = 0.001), basophils (p = 0.001), haemoglobin (p = 0.002), red blood cells (p = 0.001), platelet count (p = 0.001) and fibrinogen concentration (p = 0.001). In addition, significant increases were observed in white blood cell (WBC) (p = 0.001) neutrophil (p = 0.001) concentrations and activated partial thromboplastin time (p = 0.001). Non-significant changes were observed in monocyte, mean corpuscular haemoglobin, mean cell volume, red cell distribution width, packed cell volume, mean corpuscular haemoglobin concentration, plasma viscosity and prothrombin time values (p>0.05).

### Biochemistry blood results

**Fig 2:**
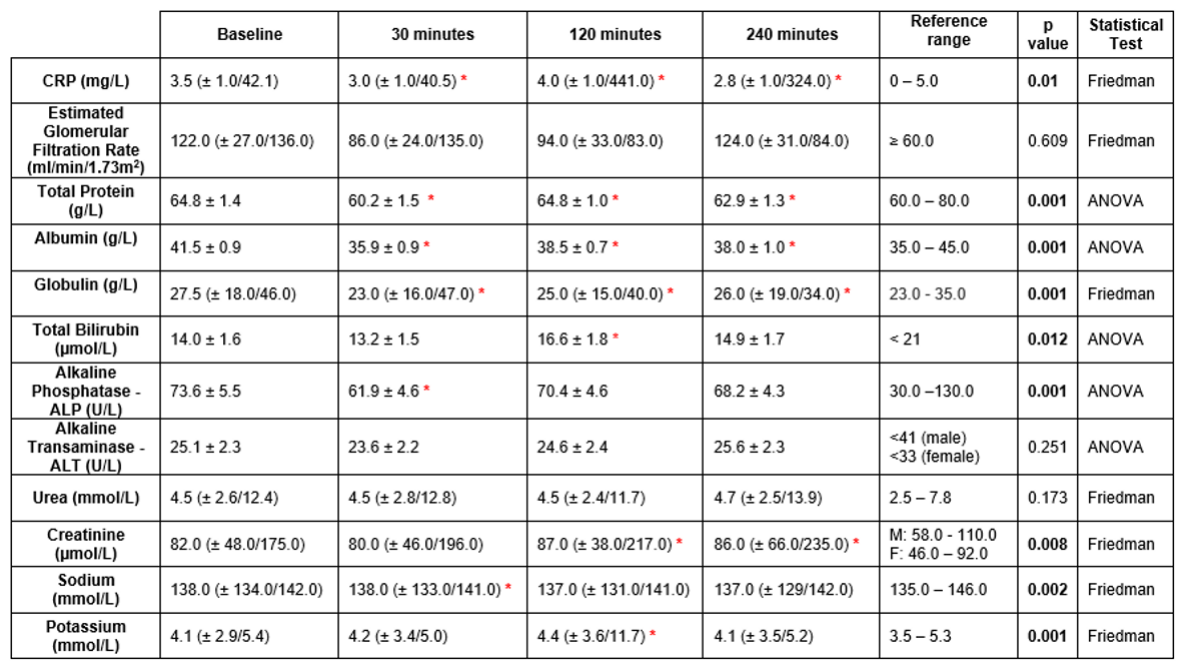
The effect of FURS, for the treatment of kidney stones on biochemistry parameters (n = 40). Data analysed via Friedman testing is presented as median ± minimum / maximum, data analysed via ANOVA analysis is presented as means ± standard error. Statistical significance following post hoc analysis is represented when*p≤0.05. (M = male, F = female).

“Fig 2” demonstrates the changes in biochemistry parameters observed following FURS for the treatment of kidney stones (n = 40). Significant decreases were observed post FURS in the following parameters: C reactive protein (p = 0.01), total protein (p = 0.001), albumin (p = 0.001), globulin (p = 0.001) and alkaline phosphatase (p = 0.001) and von Willebrand factor (p = 0.046). Whereas significant increases were observed post FURS in the following parameters: total bilirubin (p = 0.012), creatinine (p = 0.008), sodium (p = 0.002) and potassium (p = 0.001). Non-significant changes were observed in estimated glomerular filtration rate, alkaline phosphatase or urea levels (p>0.05)

### Post-operative complications

**Fig 3:**
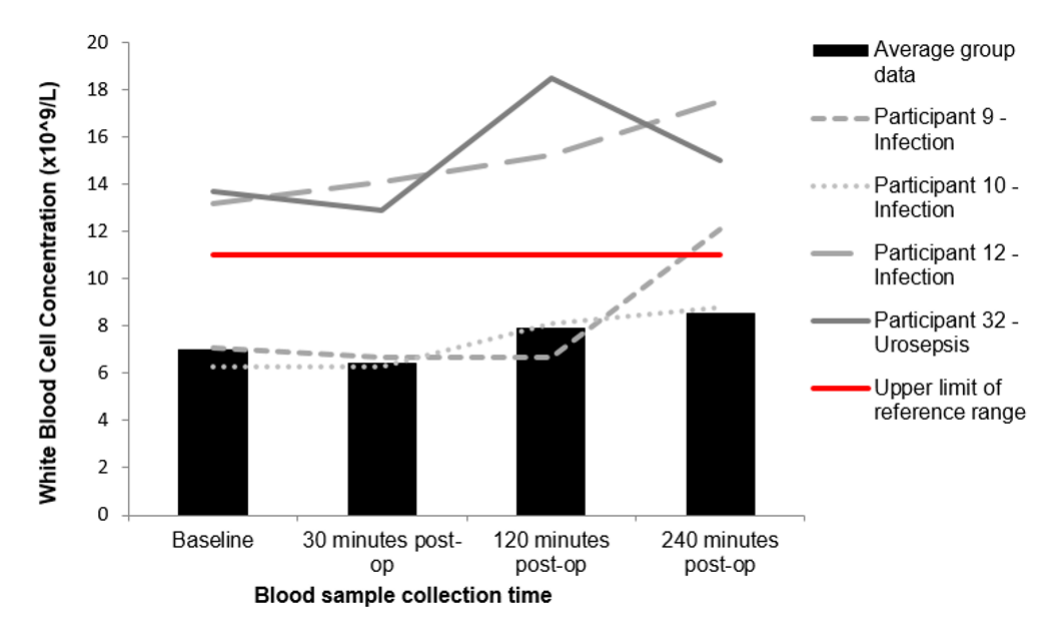
Changes to white blood cell concentrations following FURS for the treatment of kidney stone(s). Participants 9, 10, 12 and 32 who went onto develop post-operative complications compared to the average group response (n = 40). Participant 9, 12 and 32 exhibit clinically significant changes that fall outside of the normal reference range (4.0–11.0 x109/L).

**Fig 4:**
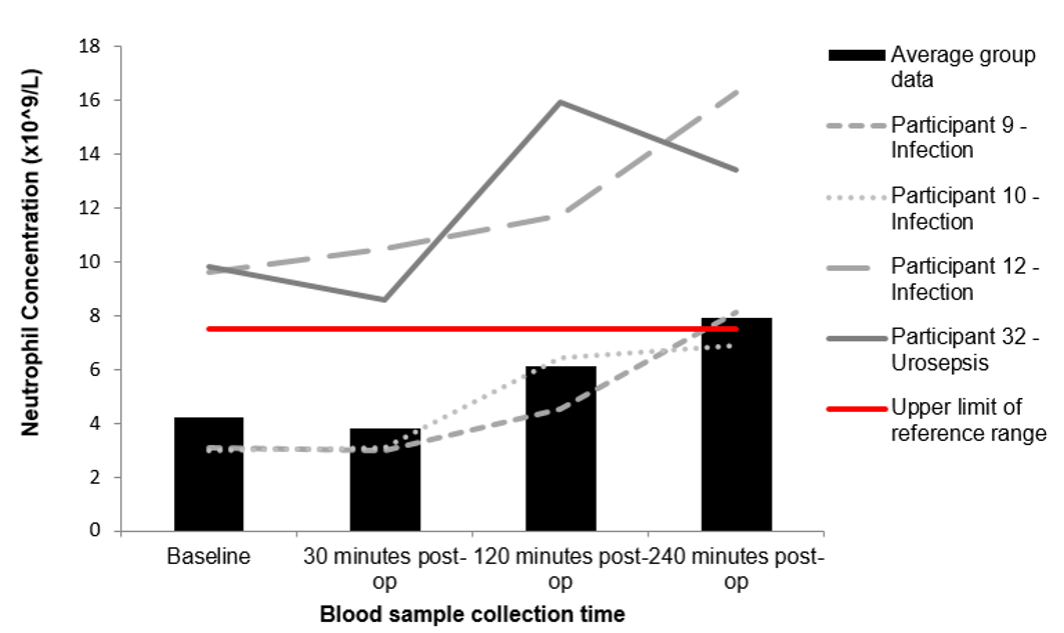
Changes to neutrophil concentrations following FURS for the treatment of kidney stone(s). Participants 9, 10, 12 and 32 who went onto develop post-operative complications compared to the average group response (n = 40). Participant 9,12 and 32 exhibit clinically significant changes that fall outside of the normal reference range (1.7–7.5 x109/L).

One female (participant 12) and two males (participants 9 and 10) developed a post-operative UTI, and participant 32 (male) developed urosepsis. “Figs 1–4” show the average results of the research population (n = 40) compared to the results from participant 9, 10, 12 and 32.

**Fig 5:**
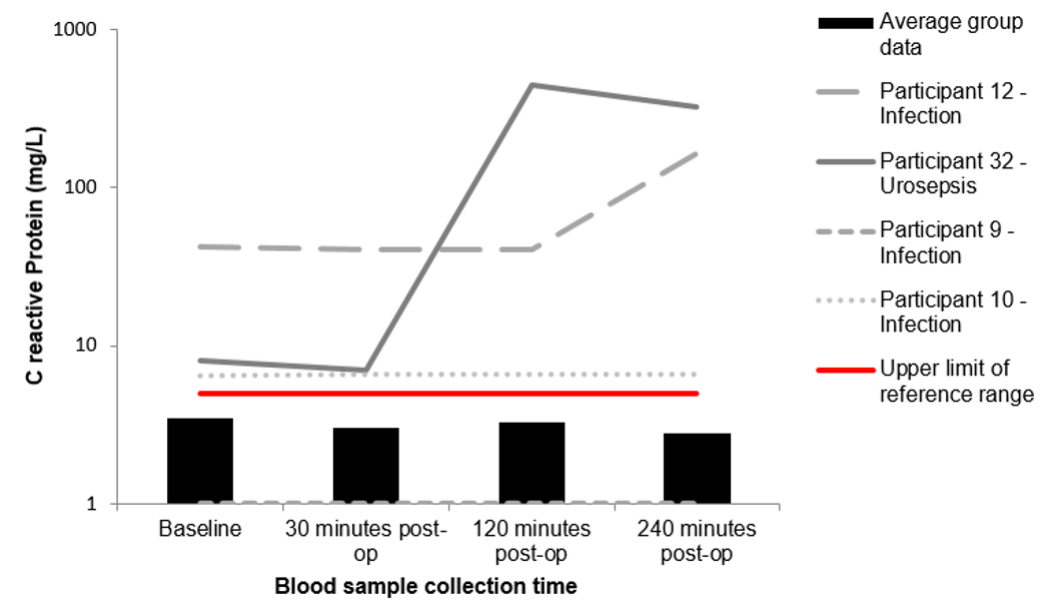
Changes to C reactive protein concentrations following FURS for the treatment of kidney stone(s). Participants 9, 10, 12 and 32 who went onto develop post-operative complications compared to the average group response (n = 40). Participant 10, 12 and 32 exhibit clinically significant changes that fall outside of the normal reference range (0–5 mg/L).

**Fig 6:**
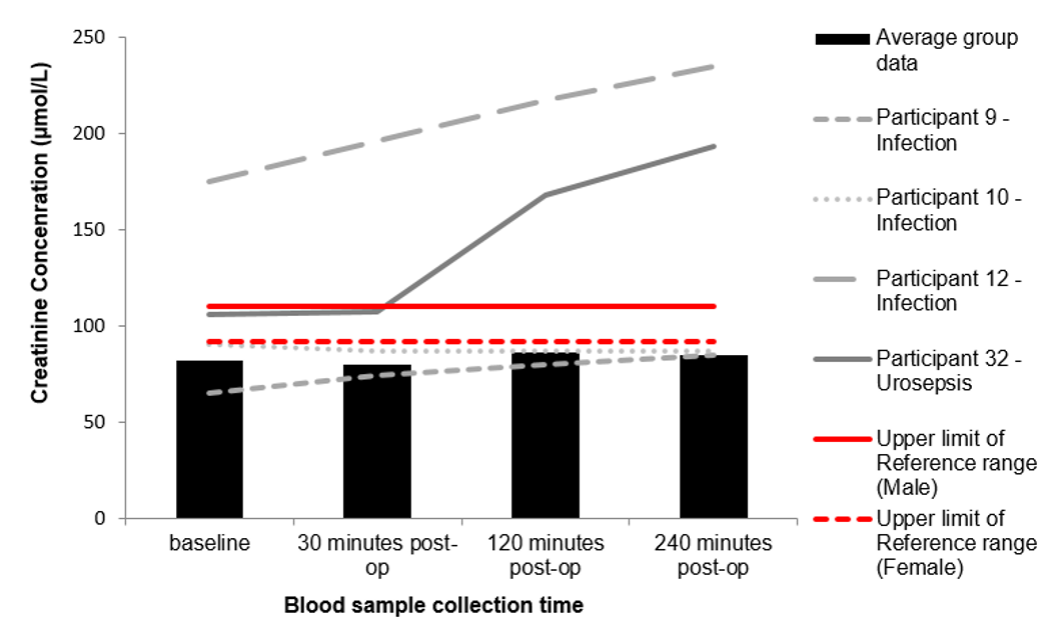
Changes to creatinine concentrations following FURS for the treatment of kidney stone(s). Participants 9, 10, 12 and 32 who went onto develop post-operative complications compared to the average group response (n = 40). Participant 9, 10, 12 and 32 exhibit clinically significant changes that fall outside of the normal reference range (male: 58–110 Mmol/L (participants 9, 10, 32), female: 46–92 Mmol/L (participant 12).

“Fig 3” shows the changes in WBC concentrations for participants 9, 10, 12 and 32 against the average group response (n = 40). Participant 12 and 32 had significantly raised WBC counts, that increased in concentration post FURS. Furthermore, at the 240 minute interval, participant 9 shows a marked increase in WBC concentration. “Fig 4” represents the changes in neutrophil concentrations following FURS for participants 9, 10, 12 and 32 against the average group response (n = 40). Participant 12 and 32 exhibit high baseline neutrophil concentrations, which rise post-operatively and peak at 240 minutes post FURS. “Fig 5” shows changes in CRP levels for participants 9, 10, 12 and 32 against the average group response (n = 40). Participant 10, 12 and 32 had CRP levels higher than the reference range at baseline, which escalated post FURS. “Fig 6” shows the changes in creatinine levels post FURS for participants 9, 10, 12 and 32 against the average group response. Participant 12 and 32 had significantly higher creatinine levels than the average group response, falling above the upper reference range limit “Fig 6”.

### Stone analysis results

Stone samples were collected during the FURS procedure from 27 participants. Post-operative calculus analysis found that 21 participants had Whewellite (CaC_2_0_4_.H_2_0 -calcium oxalate monohydrate) stones, 8 had Apatite (Ca_10_(PO_4_)_6_-(OH)_2_—calcium phosphate) stones, 4 had Cystine stones [SCH_2_CH(NH_2_)COOH]_2_, 4 had Carbonite apatite (Ca_5_(PO_3_)_3_(OH) -calcium hydroxyl phosphate) stones and 3 had Wheddelite (CaC_2_O_4_.2H_2_0—calcium oxalate dihydrate) stones.

## Discussion

The aim of this pilot-study was to investigate changes to haematological and biochemical parameters following FURS, for the treatment of kidney stones. As such, we have shown that significant changes in several haematological and biochemical biomarkers do occur.

Bleeding is a common feature following FURS, with haematuria being the most obvious manifestation. Haematuria is generally present in patients as a consequence of stone disintegration, resulting in minor lesions to the mucosa and irritation of the urothelium and damage to blood vessels and the surrounding renal anatomy [12]. Episodes of tissue trauma are linked to renal vascular damage resulting from contusion and small bleeding events, which are often localised to the renal papilla. A review by Cornu, Herrmann, Traxer and Matlaga (2016) state that the complications following endoscopic surgery are numerous, yet poorly understood due to the lack of standardisation of reporting [13]. Our results show significant changes to several parameters, including: bilirubin, albumin, aPTT, fibrinogen, platelet and RBC count which are likely to be indicative of bleeding events.

Bilirubin is a yellow pigment formed in the liver by the breakdown of haemoglobin, and its levels reflect the proportion of red blood cell haemolysis [14]. Therefore, the raised total bilirubin levels combined with the decrease in RBC counts observed in this study coincide with a haematologic decline; possibly explaining the episodes of bleeding that are seen in some participants [15]. Additionally, decreases in albumin levels, as seen in our study, may also lead to an increased risk of bleeding, as albumin functions to prevent fluid from leaking out of blood vessels.

Although we have previously reported on post-operative changes in haematological markers following various surgical procedures [16], [17] there is little evidence investigating the direct effect of FURS on haematological and biochemistry parameters. The findings from our 2015 paper evaluating haemostatic function following extracorporeal shock wave lithotripsy (ESWL) highlighted significant changes to fibrinogen and vWF concentrations following ESWL. As ESWL is a non-invasive procedure carried out with neither local nor general anaesthesia, the observed outcomes we previously documented are directly relatable to the procedure. It is envisaged that similar trends will be seen in patients undergoing FURS for the treatment of kidney stones, however it must be acknowledged that patients undergoing general anaesthesia as routine for the FURS intervention may exhibit some contributory systemic changes due to the physiological response to surgical stress or the pharmacological effects of Propofol.

The effects of anaesthesia on hemodynamic changes in patients undergoing laparoscopic vs open cholecystectomy has recently been reported by Samhan et al, (2016). Although they do not comment on routine haematological or biochemistry based parameters, their findings do report on changes seen in norepinephrine, epinephrine and cortisol; which all increase during surgery and may have an effect on haemostasis [18]. Increases in these hormone levels during surgery may have an effect on routine blood parameters. Further exploration into the effect of systemic stress following FURS may prove beneficial; with the work by Aghamir et, al (2008) evaluating inflammatory markers, concluding that changes to patients inflammatory responses can be a useful indicator for assessing post-operative complications [19]. In our study, there were significant decreases noted in fibrinogen levels at 30 and 240 minutes post-operatively (P = 0.002). It is known that during clot formation, soluble fibrinogen is converted to insoluble fibrin, which crosslinks together to stabilise clots at the site of injury. Previous studies have demonstrated that decreases in fibrinogen concentrations following cardiac surgery correlate to the amount of postoperative bleeding [20], [21]. Therefore, decreasing fibrinogen levels, coupled with the findings of prolonged aPTT times, in our study, may provide evidence to suggest that the participants in this cohort undergoing FURS may have a reduced ability to form a stable blood clot. However, as fibrinogen is a non-specific acute phase reactant, it needs to be assessed alongside other markers (ie. platelets). The results of this study show a significant decrease in platelet counts (p = 0.001) up to 2 hours post FURS, which correlate with the decrease in fibrinogen and prolonged aPTT. A likely explanation for the decrease in platelets circulating in the peripheral blood is due to redistribution to the site of trauma, where they aggregate onto the endothelium and vascular muscle lining [17]; or due to the effects of Propofol, which has been documented to have inhibitory effects on platelet aggregation in patients under general anaesthesia [22]. Similar findings were reported by Khafagy et al (2010), who observed significant post-operative changes in platelet, PT and PTT in patients undergoing general anaesthesia [23]. A decrease in vWF levels were seen post treatment in this study. Usually, one of the physiological applications of vWF is the tethering of platelets to damaged endothelial sites [24]. However, as the FURS procedure is known to cause disturbances to the normal vascular integrity, causing ureteric / renal trauma and in some instances mucosal ischaemia [25], it is thought that the release of vWF from storage organelles in the vascular endothelium may have been inhibited, therefore causing a reduction in vWF concentrations post-operatively, as seen in this study.

Leukocytes play an integral role during an inflammatory response [26]. A decrease in neutrophil concentration is recorded in this study and this may be due to the demargination and relocation of cells to the site of injury. Leukocyte demargination is known to stimulate the endothelium, resulting in the release of signalling molecules from pro-inflammatory cytokines, assisting the recruitment of additional leukocytes. This is also likely to explain the later increase in neutrophil levels seen at 120 and 240 minutes post FURS, as well as the significant changes to lymphocyte, eosinophil, and basophils that are seen in this study “Fig 1”. The results from Aghamir et al (2008) paper also reported post-operative increases (24 hours) in leukocyte concentrations, linking low- grade fever as a likely cause, which may be a contributory factor in our patient population.

One of the participants in our study who developed a post-operative infection (Participant 9), interestingly had a ‘normal’ CRP level “Fig 5”, despite CRP being clinically recognised as a marker of inflammation. As such, it may be inferred, that CRP may not always be a sensitive enough marker to detect participants as risk of developing post-operative infections, following FURS. This may be due to the nonspecific nature of CRP. Therefore, measurement of alternative markers of inflammation such as creatinine may be beneficial, as creatinine levels are regulated by the kidneys, and may provide a more relevant indicator for localised inflammation. In this study, clinically raised levels of creatinine were seen in participant 12 and 32. Overall it can be appreciated that further detailed investigations into the inflammatory process and kidney function post FURS is needed, as recent evidence links prolonged inflammation to scaring, which in combination with oxidative stress, contributes to the pathogenesis of diabetes mellitus, hypertension and diabetic nephropathy [27]. Work by Uğuz S et al, (2016) explored the effect on ozone therapy in reducing lithotripsy induced renal injury on rats during SWL and found that administering ozone resulted in significant changes to biochemical results, notably a reduction is AST and ALP, as well as ameliorating nitro-oxide stress [28]. Therefore, future monitoring for sustained inflammation combined with the potential for therapeutic intervention such as ozone therapy may lead to better patient outcomes.

To date there are very few reports on the effect of FURS, for the treatment of kidney stones, on haematology and biochemistry parameters in human subjects. We therefore believe that the results from this study are a welcome addition to the urological literature. However, in order to fully understand the changes within the peripheral circulatory system, further studies involving larger cohorts and longer post-operative monitoring and blood sampling (eg. 24hours +) is needed. The lack of a control arm in this study due to ethical restrictions is also recognised as a limitation, baseline measurements were implemented to overcome this, however sampling procedures my need some revision in future to overcome the potential effects of sampling pre-operatively whilst patients are in a non-physiological state and to fully understand the attributing effects of anaesthesia on patients’ blood parameters.

Furthermore, it is acknowledged that a limiting factor of this pilot-study is the relatively small number of participants recruited (n = 40). Nevertheless, we feel that these initial results provide encouragement for further exploration into the inflammatory and phagocytic leukocyte reactions and its correlation with clinical outcome measures (e.g. bleeding and infection), in larger cohorts following FURS. Ultimately, if changes to haematology and biochemistry markers following FURS can identify or predict those patients at increased risk, future directions of this study may offer the potential to guide practice, with the introduction of point of care testing to predict those patients at risk of complications allowing for ‘pre-emptive’ interventions to be implemented; such as pharmacological intervention using anti-inflammatory or anti-haemorrhagic agents to improve patient outcome, in those ‘at risk’ undergoing FURS for kidney stones.

## Conclusions

In conclusion, in this study we have shown several changes to haematology and biochemistry parameters following FURS. Informed examination of these parameters, in the future, could provide valuable data to aid clinicians in distinguishing a ‘normal’ response after FURS to those that are ‘abnormal’. The ultimate goal is to be able to predict those patients at risk of complications, such as bleeding or infection following FURS. The validation and reliability needs to be assessed through larger cohort studies.

## Supporting information

S1 File This file contains the raw data used in this paper. (XLSX)

## References

[1] Romero V, Alpinar H, Assimos D G. Kidney stones: a global picture of prevalence, incidence, and associated risk factors. Reviews in Urology. 2010; 12: 86–96. PMC2931286 20811557

[2] Srisubat A, Potosat S, Lojanapiwat B, Setthawong V, Laopaiboon M. Extracorporeal shock wave lithotripsy (ESWL) versus percutaneous nephrolithotomy (PCNL) or retrograde intrarenal surgery (RIRS) for kidney. John Wiley & Sons, Ltd. Cochrane Database of Systematic Reviews. 2014; 11: 4–43. 10.1002/14651858.CD007044.pub3 25418417

[3] Global Burden of Disease Study, 679 Callaborators. Global, regional, and national incidence, prevalence, and years lived with disability for 301 acute and chronic diseases and injuries in 188 countries, 1990–2013: a systematic analysis for the Global Burden of Disease Study 2013. Lancet. 2013; 386: 743–800. 10.1016/S0140-6736(15)60692-4 PMC4561509 26063472

[4] GBD Mortality and Causes of Death Collaborators. Global, regional, and national age-sex specific all-cause and cause-specific mortality for 240 causes of death, 1990–2013: a systematic analysis for the Global Burden of Disease Study 2013. Lancet. 2015; 9963, 385: 117–171. 10.1016/S0140-6736(14)61682-2 PMC4340604 25530442

[5] Portis AJ and Sundaram CP. Diagnosis and initial management of kidney stones. American Family Physician. 2001; 63,1: 1329–1338. 11310648

[6] Tuney BW, Heers H. Trends in urological stone disease: a 5-year update of hospital episode statistics. BJU International. 2016; 118: 785–789. doi: 10.1111/bju.13520 27128735 10.1111/bju.13520

[7] Tseng TY and Preminger GM. Kidney stones: flxible ureteroscopy. BMJ Clinical Evidence. 2015; 6: 89–94. PMC4632914 26535802

[8] Türk C, Knoll T, Petrik A, Sarica K, Skolarikos A, Straub M, et al Guidelines on Urolithiasis. European Association of Urology 2014: 7–94.

[9] Miller NL and Lingerman JE. Management of kidney stones. BMJ Clinincal Research. 2007; 3: 468–472. 10.1136/bmj.39113.480185.80 PMC1808123 17332586

[10] Skolarikos A, Gross A, Krebs A, Unal D, Bercowsky E, Eltahawy E, et al Outcomes of Flexible Ureterorenoscopy for Solitary Renal Stones in the CROES URS Global Study. The Journal of Urology. 2015; 194: 37–143. 10.1016/j.juro.2015.01.112 25676432

[11] McAteer J A and Evan A P. The Acute and Long-Term Adverse Effects of Shock Wave Lithotripsy. Seminars in Nephrology. 2008; 28: 200–213. doi: 10.1016/j.semnephrol.2008.01.003 18359401 10.1016/j.semnephrol.2008.01.003 PMC2900184

[12] Lingeman JE, Matlaga B, Evan AP. Surgical management of urinary lithiasis, Campbell's Urology. Philidephia: W.B Saunders Company; 2006.

[13] Cornu JN, Herrmann T, Traxer O, Matlaga B. Prevention and management following complications from endourology procedures European Urology Focus. 2016; 2: 49–59. 10.1016/j.euf.2016.03.014 28723449

[14] Lakomkin N, Sathiyakumar V, Dodd A, Jahangir A, Whiting P, Obremskey W, et al Pre-operative labs: Wasted dollars or predictors of post-operative cardiac and septic events in orthopaedic trauma patients? Injury. 2016; 47: 1217–1221. doi: 10.1016/j.injury.2016.03.004 26994519 10.1016/j.injury.2016.03.004

[15] Handa RK, Bailey MR, Paun M, Gao S, Connors BA, Willis LR, et al Pretreatment with low-energy shock waves induces renal vasoconstriction during standard SWL: a treatment protocol known to reduce lithotripsy-induced renal injury. BJU International. 2010; 103: 1270–1274. 10.1111/j.1464-410X.2008.08277.x PMC2675658 19154458

[16] Hughes SF, Hendricks BD, Edwards DR, Bastawrous SS, Middleton JF. et al Lower limb orthopaedic surgery results in changes to coagulation and non-specific inflammatory biomarkers, including selective clinical outcome measures. European Journal of Medical Research. 2013; 9: 18–40. 10.1186/2047-783X-18-40 PMC3832226 24206644

[17] Hughes SF, Thomas-Wright SJ, Banwell J, Williams R, Moyes AJ, Mushtaq S, et al A Pilot Study to Evaluate Haemostatic Function, following Shock Wave Lithotripsy (SWL) for the Treatment of Solitary Kidney Stones. PLOS one. 2015; 10: 1–9. 10.1371/journal.pone.0125840 PMC4418567 25938233

[18] Samhan Y, Radwan K, Youssef M, Ebieda R, Zeidana M, El Bendary O, et al Hemodynamic changes and stress response during BIS guided TCI anesthesia with propofol-fentanyl in laparoscopic versus open cholecystectomy. Egyptian Journal of Anaesthesia. 2016; 32(1): 45–53.

[19] Aghamir S, Mojtaba M, Meysamie A, Atharikia D, Izadpanah F, Sheikhvatan M. Comparison of systemic stress responses between percutaneous nephrolithotomy (PCNL) and open nephrolithotomy. Journal of Endourology. 2008; 22(11): 2495–2499. doi: 10.1089/end.2008.0319 19046089 10.1089/end.2008.0319

[20] Ucar HI, Oc M, Tok M, Dogan OF, Oc B, Aydin A, et al Preoperative fibrinogen levels as a predictor of postoperative bleeding after open heart surgery. The heart surgery forum. 2007; 10: 392–396. 10.1532/HSF98.20071065 17855205

[21] Ternstrom L, Radulovic V, Karlsson M, Baghaei F, Hyllner M, Bylock A, et al Plasma activity of individual coagulation factors, hemodilution and blood loss after cardiac surgery: a prospective observational study. Thrombosis Research. 2010; 126: 128–133. 10.1016/j.thromres.2010.05.028 20580414

[22] Hirakata H, Nakamura B, Yokubol H, Toda H, Hatano Y, Urabe N, et al Propofol has both enhancing and suppressing effects on human platelet aggregation in vitro. Anaesthesiology. 1991; 91: 1361–1369. 10.1097/00000542-199911000-00028 10551587

[23] Khafagy H F, Hussein N A, Radwan K G, Refaat AI, Hafez HS, Essawy FM, et al, 2010. Effect of general and epidural anesthesia on hemostasis and fibrinolysis in hepatic patients. Haematology. 2010; 15(5):360–367. 10.1179/102453310X12647083620886 20863432

[24] Iba T, Ito T, Maruyama I, Jilma B, Brenner T, Müller M, et al Potential diagnostic markers for disseminated intravascular coagulation of sepsis. Blood Reviews. 2016; 30: 149–155. doi: 10.1016/j.blre.2015.10.002 26574054 10.1016/j.blre.2015.10.002

[25] Wood K, Keys T, Mufarrij P, Assimos DG. Impact of Stone Removal on Renal Function: A Review. Reviews in Urology. 2011; 13: 73–89. 21935339 PMC3176557

[26] Butterfield TA, Best TM, Merrick MA. The Dual Roles of Neutrophils and Macrophages in Inflammation: A Critical Balance Between Tissue Damage and Repair. Journal of athletic training. 2006; 41: 457–465. 17273473 PMC1748424

[27] Arellano-Buendia A, Tostado-González M, García-Arroyo F, Cristóbal-García M, Loredo-Mendoza ML, Tapia E, et al Anti-Inflammatory Therapy Modulates Nrf2-Keap1 in Kidney from Rats with Diabetes. Oxidative Medicine and Cellular Longevity. 2016: 1–11. 10.1155/2016/4693801 PMC4756195 26955430

[28] Uğuz S, Demirer Z, Uysal B, Alp BF, Malkoc E, Guragac A, et al Medical ozone therapy reduces shock wave therapy-induced renal injury. Renal Failure. 2016; 38(6): 974–981. doi: 10.3109/0886022X.2016.1172941 27099130 10.3109/0886022X.2016.1172941

